# Sucrose non-fermenting1-related protein kinase VcSnRK2.3 promotes anthocyanin biosynthesis in association with VcMYB1 in blueberry

**DOI:** 10.3389/fpls.2023.1018874

**Published:** 2023-02-24

**Authors:** Xuan Wang, Qi Tang, Fumei Chi, Hongdi Liu, Hongjun Zhang, Yang Song

**Affiliations:** Key Laboratory of Biology and Genetic Improvement of Horticultural Crops (Germplasm Resources Utilization), Research Institute of Pomology, Chinese Academy of Agricultural Sciences, Ministry of Agriculture, Xingcheng, China

**Keywords:** SnRK2, ABA signaling pathway, fruit ripening, anthocyanin synthesis, *VcMYB1*, *Vaccinium corymbosum*

## Abstract

Sucrose non-fermenting1-related protein kinase-2 (SnRK2) is a plant-specific protein kinase family and an important component of the abscisic acid (ABA) signaling pathway. However, there is a lack of relevant studies in blueberry (*Vaccinium corymbosum*). In this study, we identified six SnRK2 family members (from *VcSnRK2.1* to *VcSnRK2.6*) in blueberries for the first time. In addition, we found that *VcSnRK2.3* expression was not only positively correlated with fruit ripening but was also induced by ABA signaling. Transient expression in blueberry fruits also proved that *VcSnRK2.3* promoted anthocyanin accumulation and the expression of anthocyanin synthesis-related genes such as *VcF3H*, *VcDFR*, *VcANS*, and *VcUFGT*. Transgenic *Arabidopsis thaliana* seeds and seedlings overexpressing *VcSnRK2.3* showed anthocyanin pigmentation. Yeast two-hybrid assays (Y2H) and Bimolecular fluorescence complementation assays (BiFC) demonstrated that *VcSnRK2.3* could interact with the anthocyanin positive regulator *VcMYB1*. Finally, *VcSnRK2.3* was able to enhance the binding of *VcMYB1* to the *VcDFR* promoter. *Via* regulation transcription of anthocyanin biosynthesis genes, *VcSnRK2.3* promoted anthocyanin accumulation in blueberry. The above results suggest that *VcSnRK2.3* plays an important role in blueberry anthocyanin synthesis, is induced by ABA, and can interact with *VcMYB1* to promote anthocyanin biosynthesis in blueberry.

## 1 Introduction

Blueberries (*Vaccinium corymbosum*, Ericaceae) are one of the most nutritious foods cultivated worldwide. In general, highbush blueberry (*V. corymbosum* L.) is considered to be a commercially important blueberry. Highbush blueberries include three types: southern highbush blueberry (SHB), half highbush blueberry (HHB), and northern highbush blueberry (NHB) (Yang et al., 2022). Blueberries are rich in flavonoids (mainly anthocyanins) and polyphenols (proanthocyanidins), which are recognized by the Food and Agriculture Organization of the United Nations (FAO) as one of the top five health foods for humans and have a promising commercial future. Anthocyanins have anti-cancer, anti-obesity, and anti-inflammatory effects, protect vision, prevent heart disease, improve immunity, and improve cognitive decline (Ma et al., 2018; Guan et al., 2021).

A change in fruit color is an important indicator of fruit maturity, which is mainly determined by anthocyanins, carotenoids and phenols (Ballester et al., 2010; Wang et al., 2020), and the anthocyanin concentration is a major factor that determines plant color. Anthocyanins are an important secondary metabolite of plants and are synthesized *via* the phenylalanine metabolic pathway (Allan et al., 2008; Jaakola, 2013; An et al., 2020a). Functionally, genes that affect anthocyanin biosynthesis are divided into two groups. The anthocyanin synthesis pathway’s essential structural genes make up one group, such as chalcone synthase (CHS), chalcone isomerase (CHI), lavanone 3-hydroxylase (F3H), dihydroflavonol 4-reductase (DFR), anthocyanidin synthase (ANS), and UDP-glucose flavonoid 3-O-glucosyltransferase (UFGT) (Boss et al., 1996; Ma et al., 2014). The other group consists of transcription factors that are not directly involved in anthocyanin synthesis but are used to regulate the expression of structural genes, such as MYB, bHLH and WD40, which usually form the transcriptional complex MYB-bHLH-WD40 (“MBW” transcription factor) (Petroni and Tonelli, 2011; Zhao et al., 2019). Previous studies have found that *MYB75*, *MYB113* and *MYB114* in *Arabidopsis* contribute to the accumulation of anthocyanins (Teng et al., 2005; Gonzalez et al., 2008; Chen et al., 2022). In apple, *MdMYB1* and its alleles play important roles in anthocyanin biosynthesis (Ban et al., 2007; Espley et al., 2007). In recent studies, *VcMYB1* was shown to function as a regulator of anthocyanin biosynthesis, and *VcMYB1* positively regulates anthocyanin synthesis and accumulation by binding to the *VcDFR* and *VcUFGT* anthocyanin biosynthesis genes’ promoters (Tang et al., 2021; Zhang et al., 2021). In addition, it has been demonstrated that the transcription factors *MYBA* and *MYBPA* co-regulate anthocyanin biosynthesis in blueberries (Karppinen et al., 2021; Lafferty et al., 2022). Although many species’ anthocyanin synthesis pathways have been examined, and attempts have been made to identify their molecular mechanisms, few relevant studies have been carried out in blueberries.

Abscisic acid (ABA) is a sesquiterpenoid compound discovered in the 1960s (Bassaganya-Riera et al., 2010). In addition to being a crucial part in plant growth, development, and stress response, ABA is also a major regulator of ripening in non-climacteric fruits (Chen et al., 2020b; Zhang et al., 2021; Gupta et al., 2022). ABA has been shown to promote ripening and anthocyanin synthesis in many non-climacteric fruits, including strawberry, pear, sweet cherry, grape, cucumber, and blueberry, as well as climacteric fruits, such as tomato, peach, mango, and melon (Karppinen et al., 2018; Oh et al., 2018). Thus, fruit ripening is significantly influenced by ABA. In a recent study, silencing of *VmNCED1*, a key gene for ABA biosynthesis, resulted in down-regulation of the expression of key anthocyanin biosynthesis genes in blueberry (Karppinen et al., 2018). Inhibiting the expression of the essential ABA biosynthesis gene *FaNCED1* in strawberries prevented ABA biosynthesis, and some fruit that was not colored could be supplemented with exogenous ABA, suggesting a molecular mechanism for the role of ABA in fruit ripening (Jia et al., 2011). Many transcription factors associated with anthocyanin accumulation and fruit ripening that are regulated by ABA have been discovered in studies. These transcription factors include MYB, bZIP, and bHLH (Shen et al., 2014; An et al., 2018; An et al., 2021). In apple, by regulating the MYB1-bHLH3 complex, *ABI5* regulates the ABA-induced anthocyanin synthesis, and ABA treatment enhances the interaction between *MdbHLH3* and *MdMYB1* (An et al., 2021). ABA signaling plays an important regulatory role in fruit ripening, but the genes and key regulators involved in its downstream regulation of non-climacteric fruit ripening and anthocyanin accumulation are poorly understood.

Reversible phosphorylation of proteins catalyzed by protein phosphatases and protein kinases is the main mechanism of post-translational regulation of protein activity and intracellular signal transduction in eukaryotes (Halford and Hey, 2009). Sucrose non-fermenting1-related protein kinase-2 (SnRK2) is one of the protein kinases involved in signal transduction in adversity (Boudsocq and Laurière, 2005). In *Arabidopsis*, the SnRK2 family contains ten members named *SnRK2.1* to *SnRK2.10* (Hrabak et al., 2003). And there are ten SnRK2 members in *Oryza sativa* and *Triticum aestivum*. *Fragaria ananassa* contains nine, *Solanum tuberosum* contains eight and *Zea mays* contains eleven (Boudsocq et al., 2004; Kobayashi et al., 2004; Han et al., 2015; Zhang et al., 2016; Bai et al., 2017). Subsequently, in many species, members of the SnRK2 family have been discovered, including *Lycopersicon esculentum* (Sun et al., 2011), *Vitis vinifera* (Boneh et al., 2012), *Malus prunifolia* (Shao et al., 2014), *Prunus avium* (Shen et al., 2017), *Musa acuminata* (Hu et al., 2017), *Pyrus bretschneideri* (Chen et al., 2020a), *Fragaria vesca* (Zhang et al., 2020).

SnRK2 is involved in osmotic stress and the ABA response and plays an important role in stress signal transduction (Yoshida et al., 2006; Huai et al., 2008; Cutler et al., 2010; Fujii et al., 2011). Studies on *Arabidopsis* have demonstrated that ABA elicits cellular responses by binding to the ABA receptor pyrabactin resistance1/PYR1-like/regulatory components of the ABA receptor (PYR/PYL/RCAR). ABA binding to the receptor protein PYR1/PYL/RCAR disables the inhibition of SnRK2 kinase by PP2C phosphatase and thus activates *SnRK2* (Cutler et al., 2010; Moreno-Alvero et al., 2017; Islam et al., 2021). Activated *SnRK2* then phosphorylates and regulates its downstream targets such as bZIP-like transcription factor *ABF*, slow anion channel 1 (SLAC1) and the MAPK signaling cascade pathway (Fujita et al., 2009; Geiger et al., 2009; de Zelicourt et al., 2016). Furthermore, *SnRK2* plays key roles in plant growth and development and a range of stress reactions (Fujii et al., 2011; Fujita et al., 2013), including the modulation of fruit development and ripening and anthocyanin biosynthesis. For example, in strawberry, virus-induced gene silencing of the ABA receptor *FaPYR1* delayed ripening and significantly decreased critical ABA signaling molecules, such as *ABI1*, *ABI3*, *ABI4*, *ABI5*, and *SnRK2s* (Chai et al., 2011). By contrast, *FaABI1* interference resulted in the overexpression of most genes associated with fruit ripening, including *ABI3*, *SnRK2s*, and *AREB1* (Jia et al., 2013). Previous studies identified *FaSnRK2.6* as a member of the SnRK2-III family, and *FaSnRK2.6* was a negative regulator of strawberry fruit ripening and anthocyanin accumulation (Han et al., 2015; Jia et al., 2022). Dehydration stress in sweet cherry resulted the expression of all *PacSnRK2* and *PacPP2C genes*, and it also promoted the accumulation of ABA and anthocyanin (Shen et al., 2017). In addition, *FvSnRK2.6* was found to mediate low-temperature-regulated anthocyanin accumulation in strawberry fruit (Mao et al., 2022).

Blueberries are rich in anthocyanins and have a high nutritional value. In this study, we selected blueberry (*V. corymbosum* “Duke”) as experimental material and identified its SnRK2 gene family members. In addition, we explored the regulatory effect of exogenous ABA on *VcSnRK2.3*. We also sought to determine the regulatory effect of *VcSnRK2.3* on blueberry and to assess whether this protein participates in the control of ripening and anthocyanin synthesis in blueberry fruit, particularly to determine its interaction with *VcMYB1*. Our data provide a novel mechanism for blueberry anthocyanin biosynthesis and could potentially lead to improved ripening practices.

## 2 Materials and methods

### 2.1 Plant materials

Nine-year-old blueberry (*V. corymbosum* “Duke”) plants collected from the small berry garden of the Fruit Tree Research Institute, Chinese Academy of Agricultural Sciences were used as experimental material. Experiments were conducted from March 2019 to February 2022 at the Ministry of Agriculture Key Laboratory of horticultural crop germplasm resources utilization, Institute of Fruit Tree Research, Chinese Academy of Agricultural Sciences (Xingcheng, Liaoning, China) and College of Horticulture Sciences, Shandong Agricultural University (Tai’an, Shandong, China). To ensure the consistency of the material, fruits were collected from the top of the inflorescence (first to mature). Samples were taken from plants under the same growth conditions for organ (root, stem, leaf, flower, and fruit)-specific expression. All samples were rapidly frozen in liquid nitrogen and stored at −80°C.

### 2.2 Gene isolation and nomenclature

Blueberry transcriptome data obtained from Illumina and SMRT sequencing were used to identify the gene family members of SnRK2 in blueberry (Tang et al., 2021). The excavated VcSnRK2 protein sequences were analyzed using the Basic Local Alignment Search Tool (BLAST) program (http://www.ncbi.nlm.nih.gov/BLAST/) in the National Center for Biotechnology Information (NCBI) database. The results showed that the six VcSnRK2 sequences had high homology with the SnRK2 protein sequences of *Solanum tuberosum*, *Arabidopsis thaliana* and *Zea mays*. The six genes were named according to the Phylogenetic Tree Construction. If VcSnRK2 clusters with *Solanum tuberosum*, it is named after the same number, including *VcSnRK2.1, 2.3, 2.4*, and *2.5*. For *VcSnRK2.2* and *2.6*, which do not cluster as closely with *Solanum tuberosum*, they are named sequentially according to their position on the chromosome in sequence.

### 2.3 Amino acid sequence analysis

The amino acid secondary structure of VcSnRK2 was predicted using the Simple Modular Architecture Research Tool (SMART) software program (http://smart.emb-lheidelberg.de/) through three rounds of PCR. The deduced amino acid sequences of VcSnRK2s were aligned using CLC Sequence Viewer 6.0 software with the default settings.

### 2.4 Quantitative real-time-PCR (qRT-PCR) analysis

RNA was extracted from different organs and different fruit stages of blueberry plants, and *Arabidopsis* leaves using TaKaRa MiniBEST Plant RNA Extration Kit (TaKaRa, Shiga, Japan). qRT-PCR was performed with *VcACTIN* as a reference gene. *Arabidopsis* polyubiquitin 10 (*AtUBQ10*) was used as a reference gene. Single-stranded cDNA was obtained using a reverse transcription kit (TaKaRa, Shiga, Japan). 4 μg RNA was used as a template for cDNA synthesis. The procedure for cDNA synthesis was to incubate the reaction solution at 65°C for five minutes, 42°C for 60 minutes, and 70°C for 5 minutes. qPCR assays were performed on a CFX Connect real-time PCR assay system (Bio-Rad, USA) using the SYBR Green I chimeric fluorescence assay. The amplification reaction procedure was as follows: Stage 1 pre-denaturation, the mixture was reacted at 95°C for 30 sec, one cycle; Stage 2 cyclic reaction, 95°C for 10 sec, 60°C for 30 sec, 40 cycles; Stage 3 melting curve, 95°C for 15 sec, 60°C for 60 sec, 95°C for 15 sec, one cycle. The qPCR results were all analyzed using the comparative CT method. Three biological replicates and three technical replicates were conducted. All primers used in this study are listed in **Supplementary Table S1**.

### 2.5 Analysis of the anthocyanin content by high-performance liquid chromatography

Extraction methods for anthocyanins were based on previous studies (Latti et al., 2010; Pertuzatti et al., 2021). Separation, identification, and quantification of anthocyanins by high-performance liquid chromatography (HPLC) was conducted on an Agilent 1100 Series system (Agilent, Germany), equipped with DAD (G1315B), and an LC/MSD Trap VL (G2445C VL) electrospray ionization mass spectrometry (ESIMSn) system that was coupled to an Agilent ChemStation (version B.01.03) data-processing station (Castillo-Munãoz et al., 2009). Mass spectra data were processed with Agilent LC/MS Trap software (version 5.3). ESI-MSn was used to determine the anthocyanin profiles and the following parameters were employed: positive ionization mode; dry gas, N2, 11 mL/min; drying temperature, 350°C; nebulizer, 65 psi; capillary, –2500 V; capillary exit offset, 70 V; skimmer1, 20 V; skimmer 2, 6 V; compound stability, 100%; scan range, 50–1,200 m/z (Pertuzatti et al., 2016). For the purposes of quantification, DAD chromatograms were extracted at 520 nm for anthocyanins.

### 2.6 Ectopic expression of VcSnRK2.3 in *Arabidopsis*

Agrobacterium tumefaciens GV3101 transformed the pRI101-AN vector with the open reading frame (ORF) after it had been cloned and inserted. Transformation of *Agrobacterium* into *Arabidopsis* by flower dip method. On MS media containing 50 mg L^−1^ kanamycin, T1 *VcSnRK2.3* transgenic plants were selected. Until seed set, kanamycin-resistant T1 seedlings were cultivated in a growth chamber at 22°C and a 16 h day length. Seeds of T1 plants were planted and germinated to obtain T2 seedlings. T2 seedlings were also selected on MS medium containing 50 mg L^−1^ kanamycin.

### 2.7 Blueberry injection assays

Blueberry fruit injection assays were performed with reference to previous studies (An et al., 2019; Tang et al., 2021). The open reading frame of the *SnRK2.3* gene was cloned into the pGreenII62-SK vector to obtain the *VcSnRK2.3*-pGreenII62-SK vector. Blueberry peels were injected with the mixed vector and *Agrobacterium* solutions.

### 2.8 Yeast two-hybrid assay (Y2H)

The Y2H experiment was performed according to Clontech’s instructions. The ORF, N-terminus, and C-terminus of *VcSnRK2.3* and the ORF of *MdMYB1* were inserted into pGAD and pGBD vectors. The plasmids were co-transformed into yeast strain Y2H Gold (TaKaRa, Beijing, China) by using the lithium acetate method with reference to Veries et al. For transformation, 4-5 μl of plasmid and 50 μg of denatured salmon sperm vector DNA are mixed. Then 500 μl of polyethylene glycol (PEG) lithium acetate solution is added. After incubation 20 μl DMSO is added and incubated again. The transformed yeast strains were grown on medium lacking Leucine and Tryptophan (SD-L/-T), Leucine, Tryptophan, Histidine and Adenine (SD-L/-T/-H/-A) or Leucine, Tryptophan, Histidine, Adenine and X-gal (SD-T/-L/-H/-A+X-gal) at 28°C for 3 days.

### 2.9 BiFC assay

The ORF and C-terminus of *VcSnRK2.3* and the ORF of *MdMYB1* were inserted into YFP^C^ or YFP^N^ to generate the *VcSnRK2.3*-C-YFP^C^, *VcSnRK2.3*-YFP^C^ and *VcMYB1*-YFP^N^ plasmids. The constructs were transformed into *Agrobacterium* GV3101. The specified plasmid-carrying *Agrobacterium* solution was incubated with prepared tobacco leaves for 30 minutes. After 2 days of darkness at 24°C, the transformed tobacco leaves were observed under a Confocal microscope (Zeiss LSM 780, Lena, Germany). The excitation wavelength of YFP is 510 nm, and the emission wavelength is 527 nm.

### 2.10 Firefly luciferase complementation assay

*VcDFR* promoter sequences were obtained from pre-lab data (Tang et al., 2021). Tobacco transient expression assays were carried out according to previous studies (Shang et al., 2010; An et al., 2017). The VcDFRpro : Luc reporter construct was created by amplifying the VcDFR promoter and cloning it into the pGreenII0800-LUC vector. The ORFs of *VcMYB1* and *VcSnRK2.3* were cloned into the pGreenII62-SK vector to generate 35Spro : *VcMYB1* and 35Spro : *VcSnRK2.3*, respectively. Transformed leaves were sprayed with 100 mM luciferin and placed in the dark for 5 minutes before being examined for luminescence. The LUC pictures were collected using a charge-coupled device-imaging apparatus (NightOWL LB983 in conjunction with Indigo software).

### 2.11 GUS staining and activity analysis

Effector constructs were generated from *Agrobacterium* GV3101 strains containing the pRI101-*VcSnRK2.3* and pRI101-*VcMYB1* genes. The reporter constructs were generated using the promoter sequences of *VcDFR* cloned upstream of the β-glucuronidase (GUS) reporter gene in the pCAMBIA1301 vector. *Agrobacterium* GV3101 strains carrying pRI101-*VcSnRK2.3* and pRI101-*VcMYB1* were co-injected into the abaxial surface of tobacco leaves. Infected leaves were grown in growth chambers for 3-4 days and then analyzed for GUS activity. Proteins were extracted from infected leaves and fluorescence was measured with a fluorometer (VersaFluor Fluorometer, Bio-Rad) (http://www.bio-rad.com) with reference to Jefferson et al., 1987 and Bowling et al., 1994.

## 3 Results

### 3.1 Genome-wide identification of VcSnRK2 subfamily members

**Figure 1:**
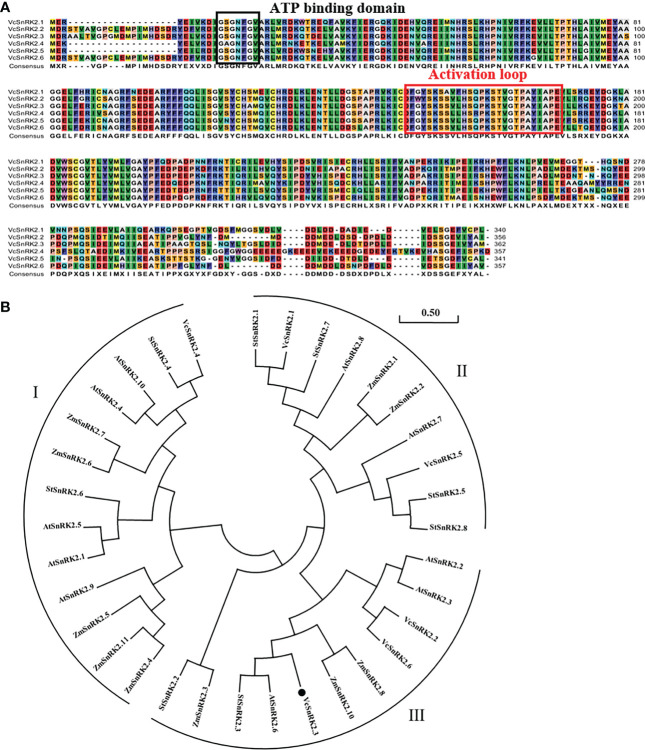
Sequence alignments and phylogenetic analysis of SnRK2.3. **(A)** Sequence alignments of the deduced amino acid sequences of *VcSnRK2.1* to *VcSnRK2.6*. The same color shading indicates the same amino acid residues. The deduced amino acid sequences of VcSnRK2s were aligned using CLC Sequence Viewer 6 software with the default settings. The predicted functional domains are boxed. **(B)** Phylogenic analysis of VcSnRK2 and its homologs in *Arabidopsis, Solanum tuberosum* and *Zea mays*. VcSnRK2.3 is denoted by the black spot. SnRK2 of *Arabidopsis, Solanum tuberosum* and *Zea mays* (AtSnRK2.1: AT5G08590.1; AtSnRK2.2: AT3G50500.2; AtSnRK2.3: AT5G66880.1; AtSnRK2.4: AT1G10940.2; AtSnRK2.5: AT5G63650.1; AtSnRK2.6: AT4G33950.1; AtSnRK2.7: AT4G40010.1; AtSnRK2.8: AtSnRK2.9: AT2G23030.1; AtSnRK2.10: AT1G60940.1; StSnRK2.1: JX280911; StSnRK2.1: JX280911; StSnRK2.2: JX280912; StSnRK2.3: JX280913; StSnRK2.4: JX280914; StSnRK2.5: JX280915; StSnRK2.6: JX280916; StSnRK2.7: JX280917; StSnRK2.8: JX280918; ZmSnRK2.1: EU676033.1; ZmSnRK2.2: EU676034.1; ZmSnRK2.3: EU676035.1; ZmSnRK2.4: EU676036.1; ZmSnRK2.5: EU676037.1; ZmSnRK2.6: EU676038.1; ZmSnRK2.7: EU676039.1; ZmSnRK2.8: EU676040.1; ZmSnRK2.10: EU676041.1; ZmSnRK2.11: EU676042.1).

Based on the blueberry transcriptome data, we obtained six blueberry SnRK2 gene family members, named *VcSnRK2.1* to *VcSnRK2.6*. We analyzed the physicochemical properties of the blueberry SnRK2 gene family members using the online software ProtParam. **Table 1** shows that the amino acid lengths of the six blueberry SnRK2 proteins, ranging from 340 (VcSnRK2.1) AAs to 362 (VcSnRK2.3) AAs, and the molecular weights ranged from 38.56 kDa (VcSnRK2.1) to 41.04 (VcSnRK2.3) kDa. In addition, the pI was in the range of 4.75 (VcSnRK2.6) to 5.62 (VcSnRK2.4). To investigate the structural features of the SnRK2 protein sequences, we conducted a comparative analysis of the amino acid sequences of the blueberry SnRK2 proteins (**Figure 1A**). The results showed that all family members contain ATP binding domains and a protein kinase activation loop and were relatively conserved (Boudsocq et al., 2004; Han et al., 2015).

**Table 1:** Characteristics of VcSnRK2 genes in blueberry (*V. corymbosum*).

Gene name	Group	Chromosome location	Size (Amino Acids)	MW (kDa)	PI	Genbank accession numbers
*VcSnRK2.4*	I	Chr9:12023838.12028814	357	40.92	5.62	OP626311
*VcSnRK2.1*	II	Chr5:7032403.7039108	340	38.56	5.59	OP626312
*VcSnRK2.5*	II	Chr8:1241970.1244296	341	38.69	5.06	OP626313
*VcSnRK2.3*	III	Chr9:21836159.21841564	362	41.04	4.79	OP626314
*VcSnRK2.2*	III	Chr5:23480352.23503781	356	40.72	4.84	OP626315
*VcSnRK2.6*	III	Chr8:14557973.14564911	357	40.68	4.75	OP626316

In addition, to investigate the evolutionary relationship between the blueberry SnRK2 gene and SnRK2 members in other plants, we downloaded the entire SnRK2 gene sequence from the NCBI website for *Arabidopsis*, *Solanum tuberosum*, and *Zea mays*. The Protein BLAST program was used to obtain homologs of *Arabidopsis*, *Solanum tuberosum* and *Zea mays* SnRK2. The evolutionary tree was constructed in MEGA-X software using neighbor- joining, which contained a total of 34 SnRK2 protein sequences. Based on the evolutionary results, *VcSnRK2.3* had the highest similarity to *StSnRK2.3* and *AtSnRK2.6* (**Figure 1B**).

### 3.2 Temporospatial expression of *VcSnRK2.3*

**Figure 2:**
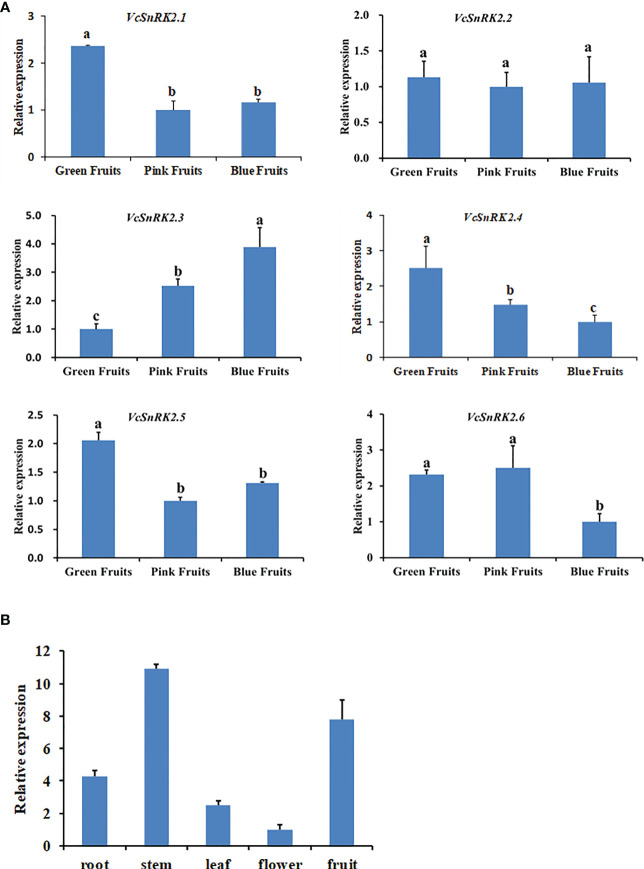
Temporospatial pattern of VcSnRK2 expression in blueberry fruits. **(A)** Quantitative real-time PCR (qRT-PCR) analysis of VcSnRK2.1 to VcSnRK2.6 expression in blueberry fruits at different developmental stages. Green fruits (40 days after flowering), pink fruits (65 days after flowering), and blue fruits (80 days after flowering). The qRT-PCR was performed with *VcGAPDH* as a reference gene. Different letters above the bars indicate a significant difference based on one-way ANOVA (*P* < 0.05). Values are the means + SD of three biological replicates. **(B)** qRT-PCR analysis of *VcSnRK2.3* expression in different organs of blueberry plants. Total RNA was isolated from organs (roots, stems, leaves, flowers and fruits). The qRT-PCR was performed with *VcGAPDH* as a reference gene. Values are the means + SD of three biological replicate.

To analyze the expression of each member of the blueberry VcSnRK2 subfamily at different fruiting stages, we examined the expression of VcSnRK2 during the green, pink, and blue fruit stages of blueberry using quantitative real-time PCR analysis (qRT-PCR). According to **Figure 2A**, the expression of *VcSnRK2.3* increased significantly when blueberry fruit ripened from the green fruit stage, indicating a possible positive regulatory relationship between *VcSnRK2.3* and blueberry fruit ripening. In contrast, the expression of *VcSnRK2.4* decreased significantly with fruit ripening, and the expression of *VcSnRK2.1* and *VcSnRK2.5* decreased slightly. For *VcSnRK2.2* and *VcSnRK2.6*, we did not observe any correlation with fruit ripening. Thus, we hypothesized that *VcSnRK2.3* could positively regulate the ripening of blueberry fruits. And further experiments were designed to verify its function.

Therefore, we investigated the distribution of *VcSnRK2.3* in different organs of blueberry, and qRT-PCR analysis was carried out on sample materials. The results showed that *VcSnRK2.3* genes were expressed in roots, stems, leaves, flowers and fruits. The expression of *VcSnRK2.3* was stem > fruit > root > leaf > flower (**Figure 2B**). Because the relative expression of VcSnRK2.3 was high in blueberry stems and fruits, we speculated that *SnRK2.3* may play a relatively important role in stems and fruits.

### 3.3 Expression of *VcSnRK2.3* in response to ABA

**Figure 3:**
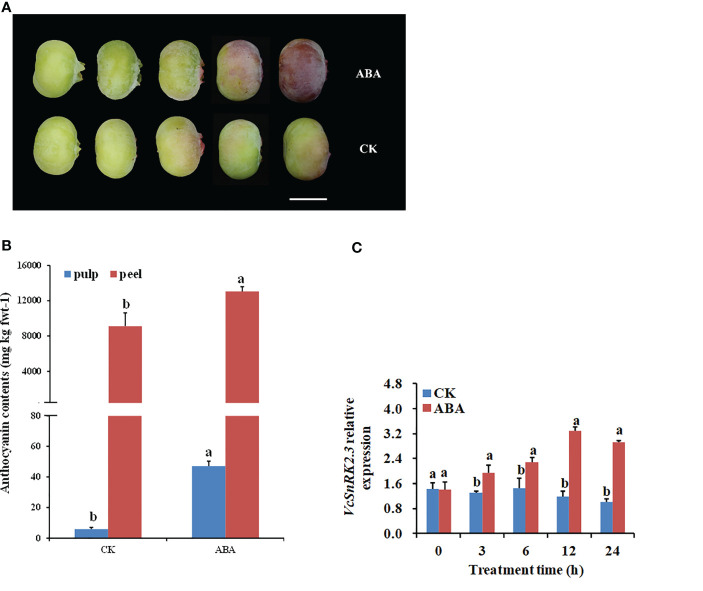
ABA and CK treatment in blueberry fruits. **(A)** Isolated blueberry fruits were treated with 100 μM ABA immersion at 0, 3, 6, 12, and 24 h. CK indicates the negative control (no treatment). Scale bar = 8 mm. **(B)** Anthocyanin contents of ripe blueberry fruits. Different letters above the bars indicate a significant difference based on a t-test (*P* < 0.05). Values are the means + SD of three biological replicates. **(C)** qRT-PCR analysis of *VcSnRK2.3* relative expression in blueberry fruits. Different letters above the bars indicate a significant difference based on a t-test (*P* < 0.05). Values are the means + SD of three biological replicates.

ABA has been shown to promote anthocyanin accumulation during blueberry fruit ripening. In our study, ABA treatment promoted the synthesis of blueberry anthocyanins. The color of blueberry fruits gradually changed from green to pink as the treatment continued within the 24 h period (**Figure 3A**). In addition, when compared to the control (CK) treatment, the anthocyanin content of ripen blueberry fruits was considerably higher following ABA treatment (**Figure 3B**). To investigate the response of blueberry *VcSnRK2.3* to ABA, we measured the expression of *VcSnRK2.3* after ABA treatment of blueberry fruits. As shown in **Figure 3C**, the control (CK) treatment had no significant effect on the expression of *VcSnRK2.3* in fruits. The expression of *VcSnRK2.3* increased gradually and then decreased slightly with the increase in the ABA treatment time, and the highest value at 12 h was 2.8 times that of the CK treatment (**Figure 3C**). The results indicated that *VcSnRK2.3* was strongly induced by ABA, which is in line with other discoveries (Boudsocq et al., 2004; Kulik et al., 2011).

### 3.4 Functional validation of VcSnRK2.3

**Figure 4:**
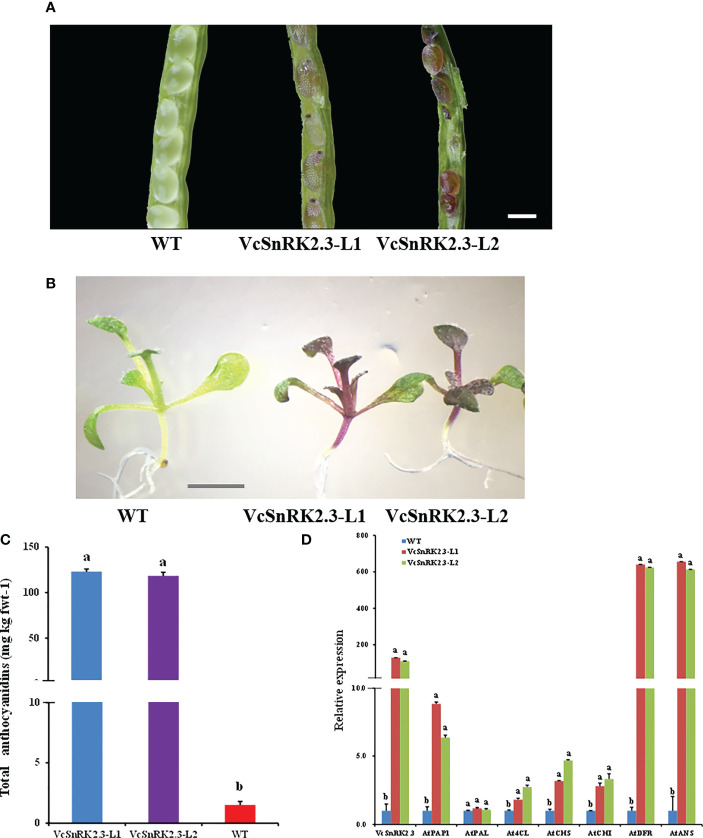
Functional analysis of *VcSnRK2.3* in a heterologous system. **(A)** Seeds of *Arabidopsis thaliana* (ecotype Columbia) plants wild-type (WT) and overexpressing 35S:*VcSnRK2.3* (*VcSnRK2.3*-L1, L2). Scale bar = 1 mm. **(B)** *Arabidopsis* plants wild-type and overexpressing 35S:*VcSnRK2.3* (*VcSnRK2.3*-L1, L2). Scale bar = 5 mm. **(C)** Anthocyanin contents of *Arabidopsis* plants overexpressing 35S:*VcSnRK2.3* (*VcSnRK2.3*-L1, L2) and the wild-type. Different asterisks above the bars indicate significant differences based on a t-test (*P* < 0.01). Values are the means + SD of three biological replicates. **(D)** Relative expression levels of *VcSnRK2.3*, *AtPAP1*, *AtPAL*, *At4CL*, *AtCHS*, *AtCHI*, *AtDFR* and *AtANS* in *Arabidopsis* plants overexpressing 35S:*VcSnRK2.3* (*VcSnRK2.3*-L1, L2) and the wild-type. All values were calculated based on the housekeeping gene *AtUBQ10*. Different asterisks above the bars indicate significant differences based on a t-test (*P* < 0.01). Values are the means + SD of three biological replicates.

In strawberry, fruit ripening and anthocyanin accumulation were shown to be negatively regulated by *FaSnRK2.6* (Han et al., 2015). To study the function of *VcSnRK2.3* and validate its effect on anthocyanin biosynthesis, we carried out heterologous expression in *Arabidopsis* (ecotype Columbia) under CaMV-35S control. Compared with the wild-type (WT), transgenic *Arabidopsis* had red pigmentation in seeds 8–10 days after flowering (**Figure 4A**). The cotyledons and hypocotyls of *Arabidopsis* seedlings grown from the above seeds were also pigmented (**Figure 4B**). The total anthocyanin content of WT *Arabidopsis* was 1.513 mg/kg, while the mean value for total anthocyanin contents of *VcSnRK2.3 Arabidopsis* (*VcSnRK2.3*-L1, L2) was 119.91 mg/kg (**Figure 4C**). In addition, RNA was extracted from the above plants and examined using qRT-PCR. The results demonstrated that overexpression of *VcSnRK2.3* up-regulated the expression of primary genes involved in the flavonoid biosynthetic pathway, including *AtPAP1, At4CL, AtCHS, AtCHI, AtDFR* and *AtANS* (**Figure 4D**). Among them, the transgenic *Arabidopsis* plants had the highest expression of structural genes *AtDFR* and *AtANS*.

**Figure 5:**
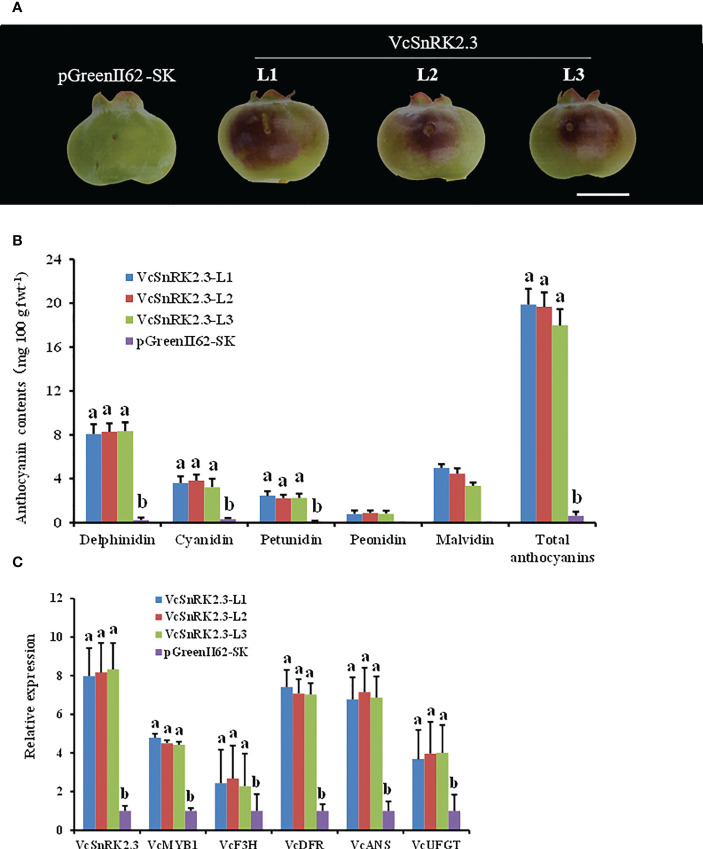
Functional analysis of Vc*SnRK2.3* in blueberry. **(A)** Blueberry fruits overexpressing pGreenII62-SK and *VcSnRK2.3*-pGreenII62-SK (*VcSnRK2.3*-L1, L2 and L3). Scale bar = 5 mm. **(B)** Anthocyanin contents of fruits overexpressing *VcSnRK2.3*-pGreenII62-SK (*VcSnRK2.3*-L1, L2 and L3) and pGreenII62-SK. Different English letters above the bars indicate a significant difference based on a t-test (*P* < 0.05). Values are the means + SD of three biological replicates. **(C)** Relative expression levels of *VcSnRK2.3, VcMYB1, VcF3H, VcDFR, VcANS* and *VcUFGT* in overexpressed *VcSnRK2.3*-pGreenII62-SK (*VcSnRK2.3*-L1, L2 and L3) and pGreenII62 -SK fruits. The qRT-PCR served as a reference gene with *VcGAPDH*. Different letters above the bars indicate a significant difference based on a t-test (*P* < 0.05). Values are the means + SD of three biological replicates.

To further demonstrate the function of *VcSnRK2.3*, we carried out *Agrobacterium*-mediated transient transformation assay in blueberry fruits 40 days after flowering. The control was the pGreenII62-SK empty vector, and the experimental group was the *VcSnRK2.3*-pGreenII62-SK vector (*VcSnRK2.3*-L1, L2 and L3). The results showed that overexpression of *VcSnRK2.3* facilitated anthocyanin accumulation in blueberry injection sites (**Figure 5A**). And the phenotype can be observed two days after gene overexpression and can last until fruit ripening. The total anthocyanin content, including the levels of delphinidin, cyanidin, petunidin, peonidin, and malvidin, was significantly increased (**Figure 5B**). In addition, compared with the pGreenII62-SK vector, the expression levels of *VcMYB1* and anthocyanin genes such as *VcF3H*, *VcDFR*, *VcANS*, and *VcUFGT* were considerably increased by the overexpression of *VcSnRK2.3* (**Figure 5C**). Therefore, based on the above two experiments, we might infer that *VcSnRK2.3* can promote not only anthocyanin biosynthesis but also the expression of *VcMYB1*.

### 3.5 Interaction analysis of *VcSnRK2.3* and *VcMYB1*

**Figure 6:**
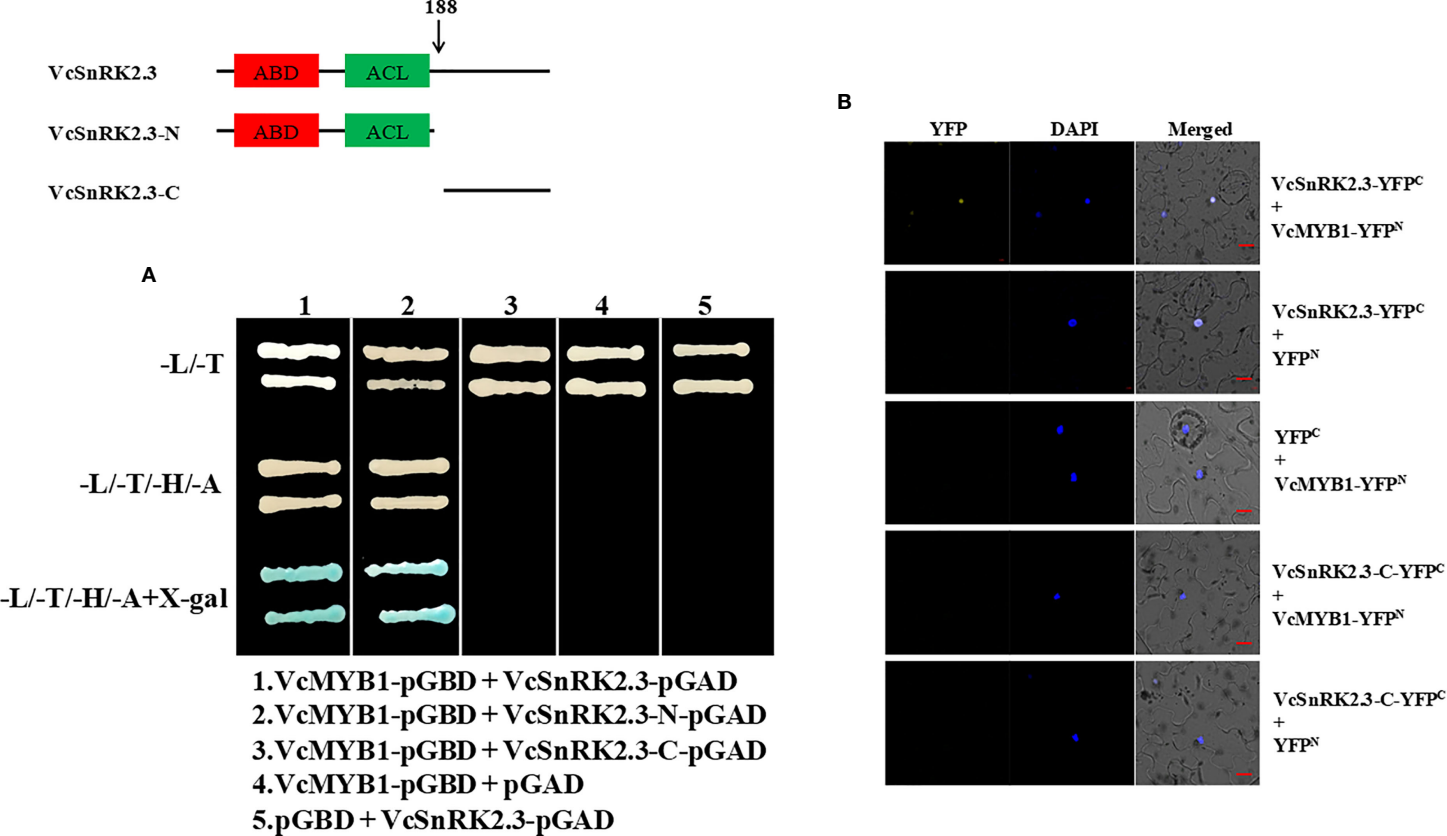
VcSnRK2.3 interacts with VcMYB1. **(A)** Yeast two-hybrid assays. The N-terminus of VcSnRK2.3 interacts specifically with VcMYB1. Yeast grown in SD-Leu/-Trp (-L/-T) medium, SD-Leu/-Trp/-His/-Ade (-L/-T/-H/-A) medium or SD-Trp/-Leu/-His/-Ade supplemented with X-gal (-T/-L/-H/-A+X-gal) medium. **(B)** Bimolecular fluorescence complementation assays. The open reading frames of *VcSnRK2.3* and *VcMYB1* were fused to the C-terminus part of YFP and the N-terminus part of YFP. Scale bar = 10 µm. A representative image was presented after this experiment was carried out three times with similar results.

The above results demonstrated that *VcSnRK2.3* can promote *VcMYB1* expression and anthocyanin accumulation in blueberry fruits (**Figure 5**). Therefore, Y2H assays were carried out to confirm the interaction between VcSnRK2.3 and VcMYB1. After transforming *VcMYB1*-pGBD and *VcSnRK2.3*-pGAD into yeast cells, the results showed that *VcMYB1*-pGBD and *VcSnRK2.3*-pGAD as well as *VcMYB1*-pGBD and *VcSnRK2.3*-N-pGAD were able to grow on SD/-Leu-Trp -His-Ade medium (**Figure 6A**). This indicated that VcSnRK2.3 interacts with VcMYB1 in yeast cells, and the action site is located at the N-terminus of VcSnRK2.3. In addition, the interaction between VcSnRK2.3 and VcMYB1 was tested by BiFC assay. As shown in **Figure 6B**, when *VcSnRK2.3*-YFP^C^ and *VcMYB1*-YFP^N^ were co-expressed, the YFP fluorescent signals could be detected. In contrast, other combinations such as *VcSnRK2.3*-YFP^C^ + YFP^N^, YFP^C^ + *VcMYB1*- YFP^N^, *VcSnRK2.3*-C-YFP^C^ + *VcMYB1*-YFP^N^, and *VcSnRK2.3*-C-YFP^C^ + YFP^N^ did not detect fluorescent signals (**Figure 6B**). Therefore, it was further demonstrated that VcSnRK2.3 and VcMYB1 interacted with each other.

### 3.6 *VcSnRK2.3* and *VcMYB1* activate *VcDFR* promoters

**Figure 7:**
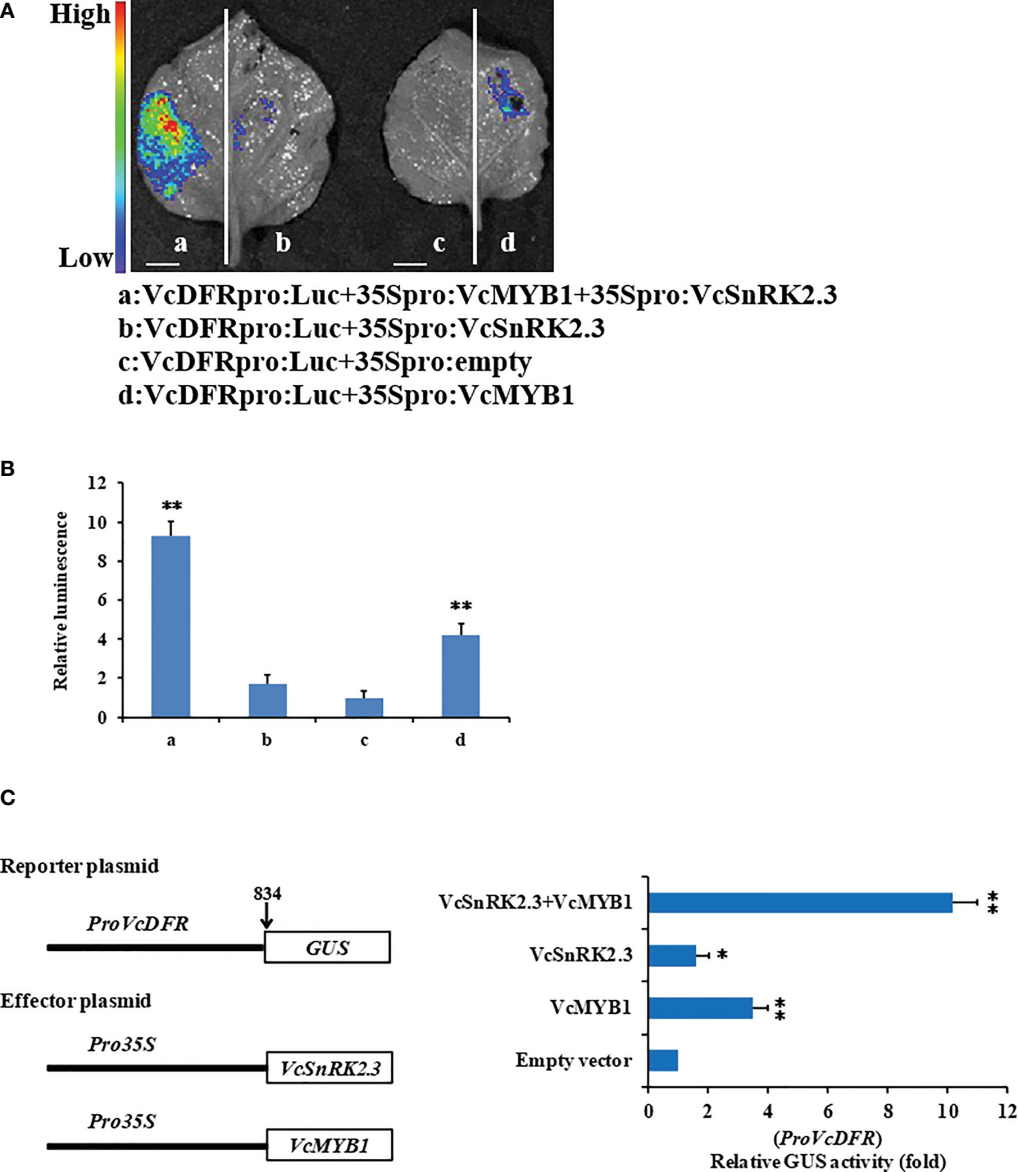
Regulation of *VcDFR*pro by *VcMYB1* and *VcSnRK2.3*. **(A)** Firefly luciferase complementation assay analysis showed that VcSnRK2.3 and VcMYB1 can activate *VcDFR* promoter activity in tobacco (*Nicotiana tabacum*) leaves. (a) *VcDFR*pro : Luc+35Spro : *VcMYB1*+ 35Spro : *VcSnRK2.3*, (b) *VcDFR*pro : Luc+35Spro : *VcSnRK2.3*, (c) *VcDFR*pro : Luc +35Spro:empty, (d) *VcDFR*pro : Luc+35Spro : *VcMYB1*. Scale bar = 10 mm. **(B)** Quantitative analysis of luminescence intensity. The value for column c (*VcDFR*pro : Luc+35Spro:empty) was set to 1. Different asterisks above the bars indicate significant differences based on a t-test (**P < 0.01). Values are the means + SD of three biological replicates. **(C)** Analysis of GUS activity. Effector vectors containing *VcSnRK2.3* and *VcMYB1* and a reporter vector containing the *VcDFR* promoter were co-injected into wild-type tobacco leaves. VcSnRK2.3 and VcMYB1 can activate *VcDFR* promoter activity. Co-injection of *VcSnRK2.3* and *VcMYB1* largely increased the activity of the *VcDFR* promoter. Empty vector used as the reference. Different asterisks above the bars indicate significant differences based on a t-test (**P < 0.01, *P < 0.05). Values are the means + SD of three biological replicates.

DFR plays a crucial role in the biosynthesis of anthocyanins. When the expression level of DFR increased, the anthocyanin content in plants increased (An et al., 2020b). A previous study showed that *MdMYB1* promotes the synthesis of anthocyanins by directly binding to the promoter sequences of *MdDFR* and *MdUF3GT* (An et al., 2018). To verify the action of the *VcDFR* promoter by *VcSnRK2.3* and *VcMYB1*, we carried out firefly luciferase complementation assay (**Figure 7A**). The results showed that the luciferase signal was highest when the *VcDFR* promoter was co-injected with *VcSnRK2.3* and *VcMYB1*, and the signal was second highest when the *VcDFR* promoter was co-injected with *VcMYB1*. Both were significantly higher than the luciferase signal of the control group (**Figure 7B**).

In a different assay, as shown in **Figure 7C**, the *VcDFR* promoter region was fused to the GUS gene as a reporter marker. The GUS activity was highest when the *VcDFR* promoter was co-injected with *VcSnRK2.3* and *VcMYB1*. And the GUS activity was also significantly higher than control when the *VcDFR* promoter was injected with *VcSnRK2.3* or *VcMYB1* alone. These results suggested that the presence of *VcSnRK2.3* up-regulated the binding of *VcMYB1* to the target promoter and activated the transcription of the anthocyanin biosynthesis genes.

## 4 Discussion

Blueberry fruits contain a large amount of anthocyanins, which are not only beneficial to human health but also improve the tolerance of plants to oxidative and drought stresses, but the biosynthesis and metabolic mechanisms of blueberry anthocyanin biosynthesis are poorly studied (Chalker-Scott, 1999; Kalt et al., 2020). Moreover, in previous studies on genes related to anthocyanin synthesis in blueberries, most of them focused on the “MBW” transcription factor and paid less attention to other genes. In this study, we identified a novel signaling component *VcSnRK2.3* that is strongly induced by ABA signaling and acts as a positive regulator of anthocyanin synthesis.

SnRK2 is a family of plant-specific protein kinases, and its members are widely distributed in plants. In this study, we identified six SnRK2 members from blueberry, and the evolutionary results showed that blueberry SnRK2 family members are divided into three subfamilies as in *Arabidopsis*, *Solanum tuberosum*, and *Zea mays* (**Figure 1B**). *OST1* is closely associated with osmotic stress in plants such as *Arabidopsis* and *Zea mays* and enhances plant drought tolerance mainly by promoting stomatal closure and reducing the rate of plant water loss (Boudsocq et al., 2004; Huai et al., 2008). In this study, we found that *VcSnRK2.3* has high homology with *OST1*. Previous studies have shown that *FaSnRK2.6*, *PacSnRK2*, and *FvSnRK2.6* can be induced by drought and temperature signals to mediate anthocyanin synthesis in fruits (Han et al., 2015; Shen et al., 2017; Mao et al., 2022). Therefore, in this study, *VcSnRK2.3* was overexpressed in *Arabidopsis*, and the results showed that the total anthocyanin content as well as anthocyanin synthesis genes increased significantly (**Figure 4**). This suggested that *VcSnRK2.3* can regulate anthocyanin accumulation in *Arabidopsis*.

In plant like apple and blueberry, the MYB1 gene has been found to promote fruit ripening and anthocyanin accumulation. It also enhances the expression of genes involved in anthocyanin synthesis, such as DFR, ANS, and UFGT (An et al., 2018; Tang et al., 2021). In our study, transient injection of *VcSnRK2.3* in blueberry fruits was able to increase the anthocyanin content and expression of genes involved in the process of anthocyanin synthesis, including *VcF3H, VcDFR, VcANS*, and *VcUFGT* (**Figure 5**). Interestingly, transient injection also increased the expression of MYB1. Therefore, we hypothesized that the promotion effect of *VcSnRK2.3* on *VcMYB1* expression is a branch of the blueberry anthocyanin biosynthetic pathway, and there is an interaction between *VcSnRK2.3* and *VcMYB1*.

Most previous studies focused on the interaction of SnRK2 with upstream signals of the ABA signaling pathway, while there are few studies on downstream transcription factors and targets. In strawberry plants, *FvMAPK3* activated acted as a downstream target of *FvSnRK2.6*, phosphorylating *FvMYB10* and reducing its transcriptional activity (Mao et al., 2022). Therefore, in this study, we explored the interaction of *VcSnRK2.3* with MYB1, a positive regulator of anthocyanin biosynthesis, as a way to enrich the mechanism of blueberry anthocyanin synthesis. Yeast two-hybrid and bimolecular fluorescence complementation analyses showed that the ATP binding domain or protein kinase activation domain of the N-terminal, the VcSnRK2.3 protein could interact with the MYB1 gene (**Figure 6**). This provided us with a new idea of anthocyanin biosynthesis in blueberry and enriches the network of anthocyanin synthesis in blueberry. In our study, MYB1 transcription factor has been shown to interact with *VcSnRK2.3* to positively regulate blueberry anthocyanin synthesis. However, the mechanism by which both regulate downstream anthocyanin synthesis-related structural genes is unclear. Therefore, we conducted further experiments to demonstrate this process.

Previous studies have shown that VcMYB1 can bind to the *VcDFR* promoter and directly control the expression of structural genes, thus, promoting anthocyanin biosynthesis (Tang et al., 2021). In blueberries overexpressing *VcSnRK2.3*, the *DFR* gene was the most differentially expressed of the anthocyanin synthesis-related genes compared to the wild type. (**Figure 5C**). Therefore, we hypothesized that DFR structural genes play a major role in regulating anthocyanin synthesis in blueberry fruits. The results showed that fluorescence and higher GUS activity were detected in the presence of *VcMYB1* alone, demonstrating that *VcMYB1* can promote anthocyanin synthesis by initiating the expression of *VcDFR*. More importantly, the fluorescence effect and GUS activity were more significant when *VcSnRK2.3* and *VcMYB1* were co-expressed (**Figure 7**). The reason is that the presence of *VcSnRK2.3* enhanced the binding of *VcMYB1* to the target promoter, thus increasing the transcriptional activity of the anthocyanin biosynthesis structural gene. In the study of *MdWRKY40* and *MdERF38, MdMYB1* not only bound to the *MdDFR* and *MdUF3GT* promoter regions, but the presence of MdWRKY40 and MdERF38 enhanced the binding of MdMYB1 to its target promoter (An et al., 2019; An et al., 2020a). This may be similar to the regulatory model of this study, where the presence of an exogenous gene regulates the transcriptional activity of MYB on the target genes, thus ultimately promoting anthocyanin accumulation in the plants.

The three key ABA receptor proteins PYR/PYL/PCAR, PP2C, and SnRK2, as well as the downstream transcription factors, make up the ABA signaling pathway. This is the more well-known signaling pathway ABA-PYR/PYL/RCARs-PP2Cs-SnRK2s (Li et al., 2016; Wu et al., 2016). In this study, we investigated the effect of *VcSnRK2.3* overexpression on blueberry anthocyanin biosynthesis and its interaction with *VcMYB1* in an attempt to find a novel pathway to regulate anthocyanin anabolism in blueberry. Previous studies on anthocyanin synthesis have focused on transcription factors such as MYB and bHLH and less on ABA signal transduction pathway genes. Therefore, through this study, we investigated the ABA signaling pathway gene *SnRK2*, thus broadening our understanding of genes related to anthocyanin synthesis. In addition, our study has enriched the knowledge of the ripening process of blueberry fruits and laid the foundation for elucidating the ripening mechanism of non-climacteric plants and other fruit trees. ABA signaling pathway genes have been identified in many plants, but there are few studies on the effects of SnRK2 downstream targets and transcription factors on anthocyanin metabolism, which may be further explored in the future.

In conclusion, we identified a new protein VcSnRK2.3 in blueberries that is involved in the regulation of blueberry anthocyanin biosynthesis. *VcSnRK2.3* expression not only positively correlated with fruit ripening but could also be induced by ABA signaling. In addition, VcSnRK2.3 can interact with *VcMYB1* to promote anthocyanin accumulation by enhancing the binding of VcMYB1 to the *VcDFR* promoter.

## Data availability statement

The datasets presented in this study can be found in online repositories. The names of the repository/repositories and accession number(s) can be found below: https://www.ncbi.nlm.nih.gov/, OP626311 OP626312 OP626313 OP626314 OP626315 OP626316.

## Author contributions

YS conceived and designed the experiments. XW, QT and YS performed the research. XW, QT, YS, FC, HL, and HZ analyzed the data. YS and XW wrote the paper. All authors contributed to the article and approved the submitted version.
