# Supplementary material for: Sucrose non-fermenting1-related protein kinase VcSnRK2.3 promotes anthocyanin biosynthesis in association with VcMYB1 in blueberry
Source: Front Plant Sci. 2023 Feb 24;14:1018874. doi: 10.3389/fpls.2023.1018874 (PMC9998538; doi:10.3389/fpls.2023.1018874)
Supplement: Supplementary file 1 [file Table_1.docx]

**Supplementary Table S1**. List of primers used in this study.

| Gene name | Primer pairs (5’-3’ Forward/Reverse) |
| --- | --- |
| VcSnRK2.1-RT-PCR  VcSnRK2.2-RT-PCR  VcSnRK2.3-RT-PCR  VcSnRK2.4-RT-PCR  VcSnRK2.5-RT-PCR  VcSnRK2.6-RT-PCR  VcMYB1-RT-PCR  VcF3H-RT-PCR  VcDFR-RT-PCR  VcANS-RT-PCR  VcUFGT-RT-PCR  VcGAPDH-RT-PCR  AtPAP1-RT-PCR  AtPAL-RT-PCR  At4CL-RT-PCR  AtCHS-RT-PCR  AtCHI-RT-PCR  AtDFR-RT-PCR  AtANS-RT-PCR  AtUBQ10-RT-PCR  VcSnRK2.3-pRI101  VcMYB1-pRI101  VcSnRK2.3- pGreenII62-SK  VcMYB1-pGreenII62-SK  VcSnRK2.3-pGAD  VcSnRK2.3-N-pGAD  VcSnRK2.3-C-pGAD  VcMYB1-pGBD  VcSnRK2.3-YFP^C^  VcSnRK2.3-C-YFP^C^  VcMYB1- YFP^N^  proVcDFR-pGreenII0800-Luc  proVcDFR-pCAMBIA1301-GUS | CCTGCCTGTTGAAGTGATGGAAG/CTATATCGGCATCATCATCCAAGTC  GAACCTCCCTGCAGACCTCATG/CAAGATCAGGATCATCGGAGTCC  GAACCTTCCGGCAGACCTGATG/CAGGTCCTCGTCCATGTCATCG  CCAAACTGCAGAGGACATCATG/GCATGTACCTCCTTAACCGTC  GAGAAGCACCCATGGTTTCTAAAG/GTCGGTATCGTCGTCAATTATGTC  GAAGAACCTGCCATCAGACTTC/CAGGATTGGAGTCCAGATCGTC  GGTGGGATGACATGTTGTTCGATTATG/GGTCCACATTGTCCATAAAAATATTAC  GACGAGCCAATCACGTTCATGGAG/CATCCCCGAAACTGTATCAACAGCC  GTCCCCACTGAGTTTAAGGGGATTCC/CGTTCCATTTCCATTTCCATTGGCAGTTG  GAGGAGCTCACCAGCATCGGCAAC/GTCCATCAGCTCTTCCGGGACGCCG  AGATAGCGGCATTAGCT/CAACCGCAGTGGTTTATG  GGTTATCAATGATAGGTTTGGCA/CAGTCCTTGCTTGATGGACC  GCTCTGATGAAGTCGATCTTC/CTACCTCTTGGCTTTCCTCT  ATCGAAGTGATCCGTTACGC/ACTCCGATTGGTGTTCCTTG  CGCAAACCCTTTCTTCACTC/ACTCCGTCGTCGTTTTGAAG  TGAGAACCATGTGCTTCAGG/CAGATGCATGTGACGTTTCC  TTTGTACCGTCCGTCAAGTC/CAATGACGGTGAAGATCACG  GTCGGTCCATTCATCACAAC/TGAGCGTTGCATAAGTCGTC  TCAAGAAAGCCGGAGAAGAG/TTGTCCACTCGCGTTGTTAG  CGTTAAGACGTTGACTGGGAAAACT/GCTTTCACGTTATCAATGGTGTCA  ggaattccatatgATGGATCGAGCTGCGCTTACGG/cgcggatccTTACATGGCATAGACTATTTC  ggaattccatatgATGATTCAATTAAAGGGTGC/cgcggatccTTACAGTACTGCTTGTTCATC  cgcggatccATGGATCGAGCTGCGCTTACGG/ccggaattcTTACATGGCATAGACTATTTC  cgcggatccATGATTCAATTAAAGGGTGC/ccgctcgagTTACAGTACTGCTTGTTCATC  ccggaattcATGGATCGAGCTGCGCTTACGG/cgcggatccTTACATGGCATAGACTATTTC  ccggaattcATGGATCGAGCTGCGCTTACGG/cgcggatccGCCATCATACTCTTTCTTGAG  ccggaattcAAGACCGCAGATGTCTGGTC/cgcggatccTTACATGGCATAGACTATTTC  ccggaattcATGATTCAATTAAAGGGTGC/cgcggatccGAAGGTTCGTGGTTGAGGCCGT  cgcggatccATGGATCGAGCTGCGCTTACGG/acgcgtcgacCATGGCATAGACTATTTC  cgcggatccAAGACCGCAGATGTCTGGTC/acgcgtcgacCATGGCATAGACTATTTC  cgcggatccATGATTCAATTAAAGGGTGCTACCG/acgcgtcgacCAGTACTGCTTGTTCATCACC  ccccctcgaggtcgaCAAATGTTTAATTGAATTATTT/tagaactagtggatcGACTGGTTCGAATATCAA  acgaattcgagctcggtaccCAAATGTTTAATTGAATTATTT/tcagatctaccatggGACTGGTTCGAATATCAA |
